# Research on the Flame Retardancy Properties and Mechanism of Modified Asphalt with Halloysite Nanotubes and Conventional Flame Retardant

**DOI:** 10.3390/ma13204509

**Published:** 2020-10-12

**Authors:** Yangwei Tan, Zhaoyi He, Xiang Li, Bin Jiang, Jiaqi Li, Yonggang Zhang

**Affiliations:** 1 National and Local Joint Engineering Laboratory of Traffic Civil Engineering Materials, Chongqing Jiaotong University, Chongqing 400074, China; 2 College of Civil Engineering, Chongqing Jiaotong University, Chongqing 400074, China; hzyzwb@cqjtu.edu.cn (Z.H.); lixianghaoyun@163.com (X.L.); hnxjiang@163.com (B.J.); 622190970103@mails.cqjtu.edu.cn (J.L.); 3 Key Laboratory of Geotechnical and Underground Engineering of Ministry of Education, Department of Geotechnical Engineering, Tongji University, Shanghai 200092, China; demonzhangyg@tongji.edu.cn

**Keywords:** asphalt binder, halloysite nanotubes, conventional flame retardant, flame retardancy properties, flame-retardant mechanism, synergistic flame retardancy

## Abstract

The inflammability of asphalt road will promote fire spread in the tunnel and produce lots of toxic smoke. To improve the fire resistance of asphalt pavement, mineral powder flame retardants are generally replaced by flame retardants in equal amounts. In this study, the effects of the synergistic flame retardancy system of halloysite nanotubes (HNTs) and conventional flame retardants (CFR) on the flame retardancy performance and mechanism of asphalt were investigated. Firstly, the flame retardancy properties of the HNTs and CFR composite modified asphalt were investigated based on the Cleveland open cup method (COC), Limiting oxygen index meter (LOI), and Cone calorimeter tests (CCTs). Then, the flame retardancy mechanism of the modified asphalt was studied based on Thermogravimetric analyzer (TGA), Fourier-transform infrared (FTIR), and Scanning electron microscopy (SEM). The results show that adding HNTs could improve the flame retardancy of the CFR modified asphalt binder. When 1 wt % HNTs and 8 wt % CFR were used, the limiting oxygen index of asphalt increased by 40.1%, the ignition temperature increased by 40 °C, while the heat release rate, total heat release, the smoke production rate, total smoke release, and other parameters decreased with varying degrees. Based on TG, FTIR, and SEM, the targeted flame retardancy mechanism and synergistic effect of HNTs/CFR flame retardancy system were revealed and summarized as three stages: (1) Stage 1, aluminum hydroxide (ATH) absorbs heat through thermal decomposition and inhibits the decomposition of lightweight components in asphalt; (2) Stage 2, aluminum diethyl phosphate (ADP) decomposes and produces organic phosphoric acid, which catalyzes crosslinking and ring thickening of asphalt and the quenching effect of phosphorus free radicals to block the combustion; and (3) Stage 3, HNTs plays an important role in increasing the integrity and density of the barrier layer. In addition, the Al_2_O_3_ produced by the decomposition of ATH, the carbon layer formed by the ADP catalyzed pitch, and HNTs play a significant synergistic effect in the formation of the barrier layer. Thus, the combination of HNTs and CFR has been proved to be a prospective flame retardancy system for asphalt.

## 1. Introduction

Due to its excellent pavement performance, asphalt pavement has become the mainstream paving form in long tunnels [1,2,3]. However, asphalt, as a kind of flammable material, is easy to be decomposed under heat in tunnel fire and produces flammable gas and toxic fumes, which further promote fire spread and hinder fire rescue and escape [4,5]. At present, the most common approach to improve the flame retardancy of asphalt is by adding flame retardants, such as phosphorus flame retardant [6,7], brominated flame retardants [8,9], inorganic compound flame retardants [10,11,12], etc. The use of CFR alone is severely limited due to their low efficiency, chemical waste, and adverse effect on pavement performance [13,14,15,16,17]. Highly efficient and environmentally friendly flame retardants have become the development trend of flame retardancy technology for asphalt.

With the development of nanotechnology, nano-clays display excellent synergistic effect with CFR in flammable polymer materials [18,19]. Therefore, the application of the nano-flame retardant system in asphalt is gaining popularity. Currently, research on the nano-layered silicate flame-retardant system is the most common. Zhang et al. [20] found that organic layered silicates (OLSs) could improve the flame retardancy performance due to their barrier and expansion property. By using CFR instead of mineral materials and nano-clay-modified asphalt, Bonati et al. [21] found that organic nano-clays could significantly improve the flame retardancy performance of asphalt. Pei et al. [22] found that there was a synergistic effect between organic montmorillonite (OMMT) and ATH, which was significant in slowing down the decomposition of asphalt binder and inhibiting smoke generation during asphalt combustion. Yang et al. [23] studied the effect of OMMT/ATH on the volatile organic compounds (VOCs) emission characteristics of asphalt during construction mixing. Liang et al. [24] studied the flame retardancy effect of organic expanded vermiculite (OEVMT) and ATH. The results show that the synergistic effect of OEVMT was more obvious compared with OMMT. Besides, it has been reported that nano-layered hydroxides (LDHs) [25,26] and expanded graphite (EG) [27,28] show excellent synergistic effects with CFR. To a certain extent, the nanolayered flame retardant system has overcome some deficiencies of CFR, and the flame retardancy effect is significantly improved with a small amount of admixture. However, poor dispersion of nanomaterials and high cost of surface modification still exist, thus it is imperative to develop a more efficient nano-flame retardancy system for asphalt.

In recent studies, halloysite nanotubes (HNTs), as a flame retardance synergistic agent, were used to improve the flame retardancy performance of polymer composites [29,30]. HNTs are a tubular nanomaterial consisting of coiled layered silicates with hydrophilic hydroxyl groups on the surface [31]. Due to their unique chemical composition and structural characteristics, HNTs contain both the excellent thermal stability of silicate materials and the good dispersibility of tubular nanomaterials. Beyond that, many studies have shown that HNTs compounding with CFR not only enhance the flame retardancy but also improve the mechanical property of composites. Li et al. [32] combined HNTs with ADP as flame retardancy for nylon 66 (PA66) and found that, when the flame-retardant system was 11 wt % ADP–1 wt % HNTs, ADP–HNTs/PA66 composite had a co-effective flame-retardant effect. The tensile strength and elongation at breakage increased with the increase of HNTs in the ADP–HNTs flame-retardant system. Lyu et al. [33] combined 6 wt % HNTs with 4 wt % 2-carboxy ethyl phenyl phosphonic acid (CEPPA) and applied it to epoxy resin flame retardant modification. They found that the heat release decreased by about 38.7%, and the tensile strength and impact strength increased by 19.4% and 17.3%, respectively. Furthermore, HNTs have good dispersibility in asphalt and need no further modification, thus they are suitable as a synergist for the modified asphalt. However, due to the complex composition of asphalt [34,35], research on the flame-retardant properties and mechanism of HNTs combined with CFR-modified asphalt binder has been less favored.

In this work, the modified asphalt was produced based on the composite of HNTs and Styrene-butadiene-styrene (SBS) modifier, and flame retardancy ADP and smoke suppressor ATH were added to prepare the flame-retardant-modified asphalt binder. Through the Cleveland open cup method (COC), Limiting oxygen index meter (LOI), and Cone calorimetric tests (CCTs), the flame retardant and smoke suppression properties of composite flame retardance modified asphalt binder were tested. Based on the thermogravimetric analysis (TGA), Fourier transforms infrared spectroscopy (FTIR), and scanning electron microscope (SEM), the flame-retardant mechanism of the HNTs and CFR modified asphalt binder was analyzed.

## 2. Materials and Methods

### 2.1. Materials

**Figure 1:**
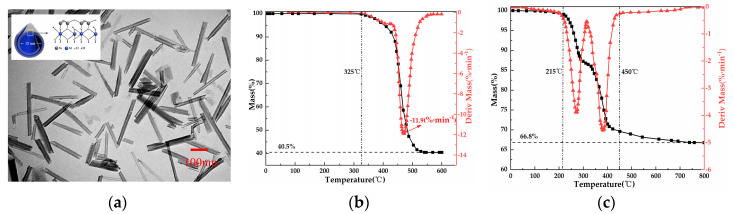
Properties of flame retardants: (**a**) SEM image of HNTs; (**b**) TGA-DTG curves of ADP; and (**c**) TGA–DTG curves of ATH.

The matrix asphalt, 70# asphalt (based on penetration), was produced by Chongqing Heavy Traffic Renewable Resources Development Co. Ltd., Chongqing, China. The physical properties of the asphalt are listed in Table 1. The flame retardant, namely ADP, was produced by Guangdong Wengjiang Chemical Reagent Co. Ltd., Guangzhou, China. The ADP is pure white powder at room temperature, the molecular weight is 119.21, and the purity is ≥98.0%. The smoke suppression agent, namely ATH, was produced by the Shandong Taixing New Material Co. Ltd., Qingdao, China. The ATH was a white power with an average particle size of 1.6–2.6 um, density of 2.4 g/cm^3^, and purity of 99.0%. The flame retardance synergist, namely HNTs, was purchased from Yuan Xin Nano Technology Co. Ltd., Guangzhou, China. The properties of flame retardants are shown in Figure 1.

According to previous studies [7,12], when CFR is compounded at 8 wt %, it can meet the requirements of flame retardancy performance and good economy. The minimum dosage of ATH is 3% as the smoke suppression is not obvious with a dosage of ATH less than 3% [11]. In polymer materials, HNTs can be combined with flame retardants under the condition of lower content (<2 wt %) and have good flame-retardant performance. Thus, 1% and 2% HNTs were selected as CFR synergistic agents, respectively [32].

### 2.2. The Preparation of Flame-Retardant-Modified Asphalt

**Figure 2:**
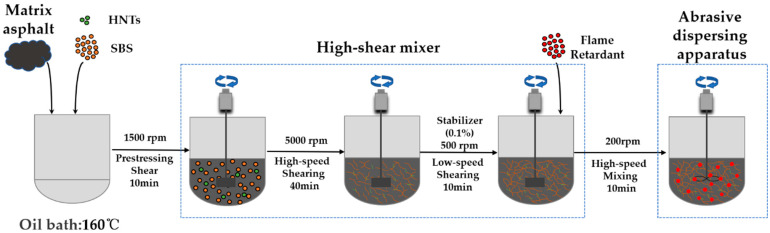
Preparation flow chart of flame-retardant-modified asphalt binder.

The flame-retardant asphalt binder was prepared by the melting method. Firstly, matrix asphalt was loaded into a stainless-steel container and heated to 160 ± 5 °C through an oil bath. Then, the SBS modifier and HNTs were added to the matrix asphalt that was stirred in an AE500S-H high-shear machine with a shearing rate of 1500 rpm for 10 min and 5000 rpm for 40 min to ensure a uniform distribution of the HNTs and SBS modifier. Finally, the mixture was stirred for an additional 10 min at 500 rpm to eliminate air bubbles, and 0.1% stabilizer was added to avoid the segregation of SBS and HNTs. To greatly simulate the field process of the dry mixing method for blending asphalt mixture, the CFR was added to BGD 750 shear abrasive dispersing apparatus and stirred at 160 °C and 200 rpm for 5 min to form the HNTs/CFR-modified asphalt binder, as shown in Figure 2. The technical indices of asphalt meet the requirements of JTG E20-2011 “Test Specification for Asphalt and Asphalt Mixture for Highway Engineering”, as shown in Table 1:

### 2.3. Testing Methods

#### 2.3.1. Testing of Flame Retardancy Properties

The ignition temperature (IT) of asphalt was conducted by COC method (Reep Instrument Co., Ltd., Zhengzhou, China) according to AASHTO T48. It refers to the ignition temperature of asphalt and is used to estimate the inflammability of asphalt.

The limiting oxygen index (LOI) testing of asphalt was conducted using a JF-3 LOI analyzer (Sanzhong Analysis Instrument Co., Ltd., Chongqing, China) according to ASTM D-2863-77. The oxygen content of asphalt burning under the limit oxygen concentration is LOI, which is used to evaluate the ability of asphalt burning continuously. The sample size was 100 mm × 10 mm × 5 mm. To prevent asphalt melting flow, glass fiber was used as the substrate to adsorb asphalt to prepare the sample.

The combustion performance of asphalt was tested by CCTs (Fire Testing Technology, London, UK) according to ISO 5660-1:2002 standard procedures under a heat flux of 50 kW/m^2^. The sample size was 100 mm × 100 mm × 5 mm. The combustion characteristics of asphalt under the real fire condition were described by heat release rate, smoke production rate, and other parameters.

#### 2.3.2. Testing of the Flame-Retardant Mechanism

An STA 449 F5 Jupiter^®^ thermogravimetry and differential scanning calorimetry instrument (NET-ZSCH Group, Bayern, Germany) was used to study the thermal properties of the flame-retardant asphalt binder. Approximately 5 mg of the sample was placed in an aluminum oxide crucible. The sample was heated from indoor temperature to 800 °C at a heating rate of 20 °C/min under the air atmosphere with a gas flux of 40 mL/min.

FTIR was recorded on a Nexus infrared spectrometer (Thermo Nicolet6700, Waltham, MA, USA) in the range of 4000–400 cm^−1^. Flame-retardant asphalt binder was treated by muffle furnace at 200, 400, 600, and 800 °C. The asphalt at 200 and 400 °C was prepared by CHCl_3_ solution, and the asphalt at 600 and 800 °C was prepared by potassium bromide pressed-disk technique.

The surface morphologies were measured by SEM (Hitachi, Ibaraki Prefecture, Japan). The samples were heated in the muffle furnace at 800 °C for 15 min, then sprayed with a thin layer of gold to improve the conductivity before SEM observation.

## 3. Results and Discussion

### 3.1. Test and Analysis of the Flame Retardant Property

#### 3.1.1. Flammability: IT and LOI

**Figure 3:**
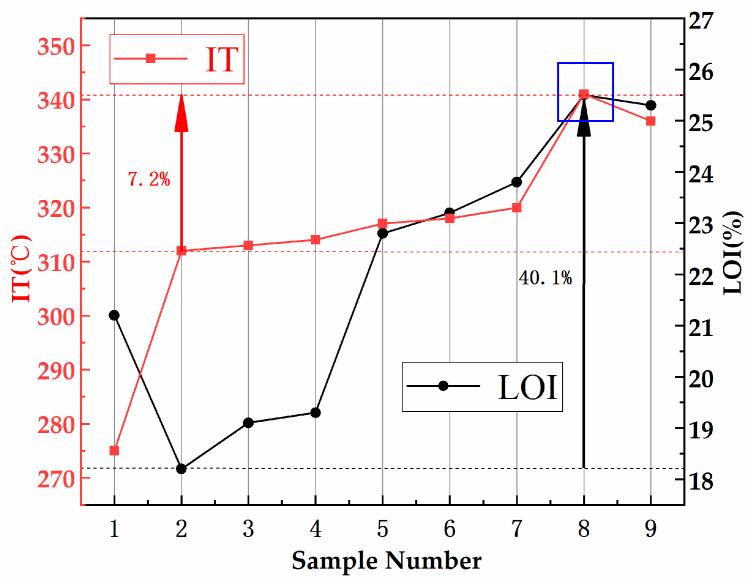
The value of IT and LOI of asphalt binder.

The IT and LOI tests are the basic methods to detect the flame retardancy of asphalt binder [36]. As shown in Figure 3, the LOI of Samples 1 and 2 are 21.2% and 18.2%, respectively, indicating that they are flammable substances and are very likely to be ignited in nature. According to Samples 3 and 4, the enhancing effect of HNTs on the value of IT and LOI of asphalt is minimal, which suggests that HNTs as flame retardant alone cannot improve the flame-retardant performance of asphalt significantly. LOI and IT are used to determine the optimal doses of ADP and ATH, which are 5 wt % and 3 wt %, respectively. The use of 8 wt % CFR (5 wt % ADP and 3 wt % ATH) alone is just enough to meet the requirements of the basic flame-retardant performance. When 1 wt % and 2 wt % HNTs were added to the 8 wt % CFR asphalt flame retardant system, respectively, and it was found that the value IT and LOI of HNTs/CFR asphalt flame retardant system increased significantly, which indicates a good synergistic effect. Combined with the conventional physical properties of asphalt binder, it was found that 1 wt % HNTs and 8 wt % CFR asphalt flame retardant systems provide the best comprehensive performance. This can be explained by the formation of the network structure of HNTs in asphalt, which makes it difficult to decompose and evaporate for the lightweight components of asphalt, such as saturated and aromatic ones. The decomposition and volatilization of these lightweight components is the key to the ignition of asphalt binder.

#### 3.1.2. Dynamic Flammability: CCTs

**Figure 4:**
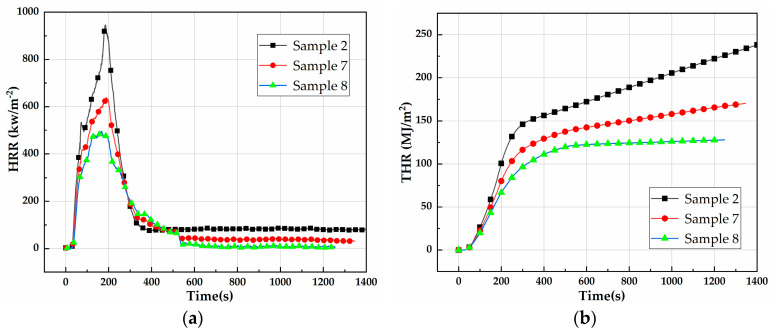
HRR (**a**) and THR (**b**) curves for asphalt binder.

**Figure 5:**
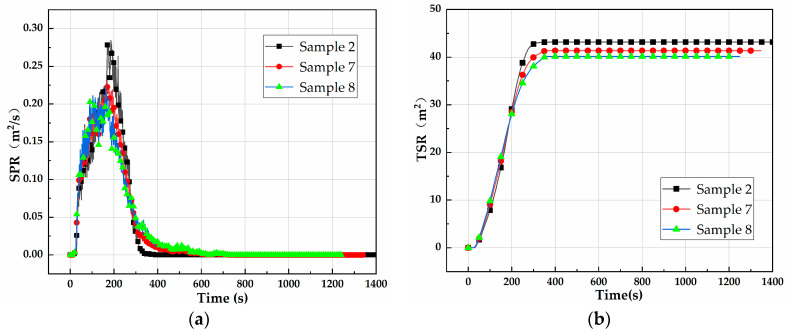
SPR (**a**) and TSR (**b**) curves for asphalt binder.

It is well known that the CCTs have a good correlation with the actual combustion on site, and CCTs are an effective method to evaluate the combustion performance of flame-retarding materials [37]. Thus, CCTs were adopted to analyze the combustion behavior of asphalt. The various parameters of CCTs at a heat flux of 50 kw/m^2^, such as time to ignition (TTI), the peak of heat release rate (PHRR), the peak of smoke produce rate (PSPR), total heat release (THR), total smoke production (TSR), and mass loss (ML), are summarized in Table 2. The HRR, THR, SPR, and TSR curves are presented in Figure 4 and Figure 5.

As shown in Figure 4a, when the heat flux value is 50 kW/m^2^ and the TTI value is 21 s, Sample 2 burned very quickly, with 993.1 kW/m^2^, which further indicates the flammability of asphalt binder in nature. The PHRR value of Sample 7 decreases to 666.6 kW/m^2^, which illustrates that the CFR-modified asphalt (Sample 7) can greatly reduce the PHRR value of asphalt binder. Besides, the TTI value appears at 28 s due to the improvement in the thermal stability of asphalt. Surprisingly, the incorporation of HNTs into the CFR system (Sample 8) further lowers the PHRR to 495.4 kW/m^2^. When the temperature exceeds 525 °C, the HRR of Sample 8 is almost zero, but the HRR of Samples 2 and 7 remains about 70 and 30 kW/m^2^, respectively.

Figure 4b shows THR for Samples 2, 7, and 8. The slope of THR curves can be assumed as the spread velocity of the fire. It is obvious that the spread velocity of Samples 7 and 8 decreases, and the flame spread velocity of Sample 8 is the lowest. When the temperature exceeds 525 °C, the flame spread of Sample 8 is almost zero, which indicates that Sample 8 may burn for 525 s and then extinguish naturally. The THR within 1400 s decreases by 28.78% with CFR and by 46.42% with HNTs/CFR compared with Sample 2, which suggests a significant effect of HNTs/CFR on flame retardance in asphalt.

Figure 5a shows that, with the addition of CFR and HNTs/CFR, the SPR and TSR of asphalt have the same trends as HRR and THR in Figure 4. When 8 wt % CFR is incorporated into the matrix asphalt (Sample 7), PSPR decreases from 0.279 to 0.231 m^2^/s compared with Sample 2. When 1 wt % HNTs/8 wt % CFR is added to the asphalt (Sample 8), PSPR further drops to 0.211 m^2^/s. At the same time, the TSR also decreases, as shown in Figure 5b. The TSR of Sample 7 decrease by 4.40% with CFR and by 7.18% with HNTs/CFR compared with Sample 2.

In conclusion, the flame-retardant properties of asphalt binder are greatly improved by the incorporation of CFR and HNTs. Moreover, the synergism effect between CFR and HNTs can greatly reduce the PHRR, HRR, PSPR, TSR, and ML values, which proves that the combustion of asphalt is drastically suppressed in the real fire. The synergistic flame-retardant system of 1 wt % HNTs and 8 wt % CFR can improve the flame retardancy performance of asphalt. The flame-retardant mechanism of the HNTs/CFR flame-retarding system is depicted in detail in the next section.

### 3.2. Flame-Retardant Mechanism

#### 3.2.1. Thermal Degradation Behavior of Asphalt Binder

**Figure 6:**
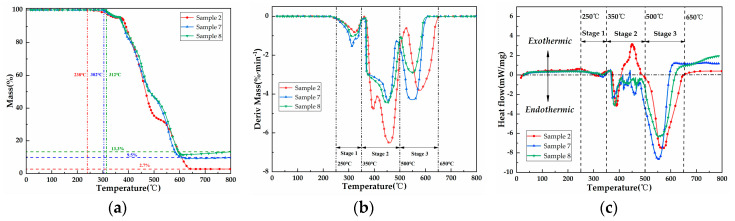
Results of TGA, DTG, and DSC test of asphalt binder: (**a**) TGA; (**b**) DTG; and (**c**) DSC.

To explore the flame retardancy mechanism of the modified HNTs and CFR asphalt binder, the thermogravimetric behavior of asphalt binder in the air atmosphere was systematically examined by TGA-DSC analysis, as shown in Figure 6.

The TGA curves of Samples 2, 7, and 8 are shown in Figure 6a. The initial decomposition temperature (T_on_, 2% weight loss) of the three samples is 238, 302, and 312 °C. The final residue generation rates of the samples are 2.7%, 9.5%, and 13.3%. The addition of flame retardants improves the initial decomposition temperature and residue generation efficiency of asphalt. However, the changes of T_on_ value for Samples 7 and 8 are not obvious. It is believed that the combination of HNTs and CFR has little effect at the early stage of asphalt combustion, and obvious synergistic effects occur at the later stage of combustion.

According to the DTG curve of asphalt thermal oxygen decomposition in Figure 6b, the asphalt binder undergoes three-step thermal degradation processes: Stage 1 at 250–350 °C, Stage 2 at 350–500 °C, and Stage 3 at 500–650 °C. In Stage 1, the lightweight components of asphalt binder are decomposed by heat. Separation and combustion of the lightweight components and decomposition and condensation of the heavy components in the asphalt binder take place mainly in Stage 2. In Stage 3, further combustion and carbonization of heavy components take place. It can be found that the addition of CFR accelerated the decomposition rate of Sample 7 in Stage 1. This may be the result of ATH decomposition under heat, which further induces heat absorption and flame retardancy. However, due to the presence of HNTs, the decomposition rate of Sample 8 decreases slightly, which may be related to the cross-linking network in the asphalt. The addition of CFR and CFR/HNTs reduces the decomposition rate of Samples 7 and 8 in Stage 2, which may be caused by the decrease in the amount of VOCs generated in the early stage. Compared with Sample 7, the decomposition rate of Sample 8 decreases from −4.29% min^−1^ to −2.89% min^−1^ in Stage 3, which suggests that HNTs have a significant enhancement effect on the thermal stability of the residue.

Similarly, the DSC curve is also divided into three stages, as shown in Figure 6c. Stage 1 is the heat absorption process, where the thermal decomposition of the light components of the asphalt mainly occurs and the ATH endothermic flame retardancy is obvious. In Stage 2, Sample 2 shows obvious exothermic phenomenon, but Samples 7 and 8 have no obvious exotherm due to the effect of flame retardant. In Stage 3, the sample shows an obvious endothermic phenomenon, which may be the result of the continuous endothermic decomposition of the residue. However, the endothermic heat of Sample 8 is significantly reduced, which may be due to the blocking effect of the residue or because the thermal stability of the residue prevents the asphalt from decomposition and burn.

According to the TGA-DTG-DSC analysis, the targeted flame retardancy mechanism and synergistic flame-retardant mechanism of CFR/HNTs are discovered. According to previous studies, it is found that the decomposition temperature zone of flame retardants is consistent with the rapid thermal weight loss stage of asphalt. It is believed that the realization of flame retardants at different combustion stages is the combined effect of the different components in the flame retardants. For example, the endothermic flame retardant of ATH takes the dominant effect in Stage 1; in Stage 2, the ADP has the flame-retardant effect of the condensed phase; and, in Stage 3, the synergistic enhancement effect of HNTs on the residue improves the flame-retardant properties of asphalt.

#### 3.2.2. FTIR Spectra of Residue for Asphalt Binders at Different Temperatures

**Figure 7:**
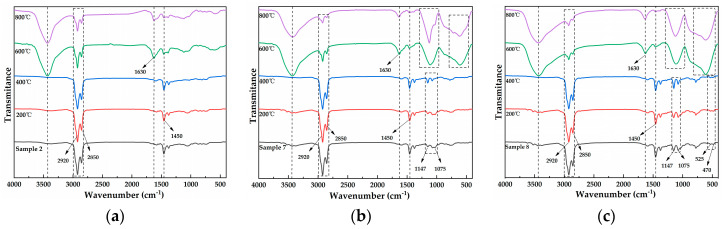
FTIR spectra of residue for asphalt binder at different temperatures: (**a**) Sample 2; (**b**) Sample 7; and (**c**) Sample 8.

To explore the flame-retardant mechanism of the synergistic flame-retardant system, the residues of the CFR/HNTs system at different temperature were characterized by FTIR, and the reaction mechanism of the flame-retardant system was studied. FTIR of Samples 2, 7, and 8 after heat treatment at 200, 400, 600, and 800 °C for 10 min are shown in Figure 7.

As shown in the FTIR spectrum, the characteristic absorption peaks of asphalt, ATH, ADP, and HNTs are: for asphalt, 2920 cm^−1^ (–CH_3_), 2850 cm^−1^ (–CH_2_–), and 1450 cm^−1^ (C–C); for ATH, 3435 cm^−1^ (–OH); for ADP, 1147 cm^−1^ (P–O) and 1075 cm^−1^ (P=O); and, for HNTs, 525 cm^−1^ (Al–O) and 470 cm^−1^ (Si–O–Si). When the temperature is 200 °C, the FTIR spectrum of Samples 2, 7, and 8 does not change significantly, which indicates that SBS-modified asphalt binder, CFR-modified asphalt binder, and CFR/HNTs system-modified asphalt binder are stable without thermal decomposition below 200 °C. When the temperature reaches 400 °C, the characteristic peak at 3435 cm^−1^ is weakened, which shows that the bound water of ATH is decomposed in asphalt. Meanwhile, the characteristic peak of 1075 cm^−1^ (P=O) in ADP is weakened, but the characteristic peak of 1147 cm^−1^ (P–O) still exists, which indicates that the phosphoric acid originated from the pyrolysis of ADP remained in the condensed phase. When the asphalt binder is heated to 600 °C, it can be found that many residues have been formed on the asphalt surface. Thus, only the characteristic peaks of 1630 cm^−1^, 1300–1000 cm^−1^, and 950–420 cm^−1^ are further described, and the influence of sampling method is ignored. The formation of 1630 cm^−1^ (C=O) may be due to the oxidation of asphalt and the dehydration of asphalt under the action of organic phosphoric acid. The peak at 1300–1000 cm^–1^ (P–O–C) is mainly formed by dehydrogenation and esterification of organic phosphoric acid with asphalt, which is an important component of the residues. Meanwhile, the big peak of 950–420 cm^−1^ can be assigned to the metal oxides containing Al and Si which are generated by HNTs/ATH and macromolecular saturated hydrocarbons which originate from asphalt polycondensation at high temperature. As the temperature rises to 800 °C, the intensity and value of most characteristic peaks are not change significantly. The increase in peak strength of 1300–1000 cm^–1^ is due to the formation of phosphate in the residue. The decrease in peak strength of 950–420 cm^−1^ may be due to the further decomposition and oxidation of the polymer in the residue.

According to the above analysis, from the perspective of the material composition of asphalt at different temperatures, the change of the peak intensity at positions 3435 cm^−1^, 1147 cm^−1^, and 1075 cm^−1^ with temperature confirms the targeted flame-retardant effect of CFR, which is mainly through the thermal decomposition of ATH to increase the decomposition temperature and reduce VOCs emissions. The catalytic flame-retardant effect of ADP in the condensed phase has been confirmed. The synergistic flame-retardant effect of HNTs is mainly to increase the content of Si-O and Al-O in the residue and increase the thermal stability. It is beneficial to form a barrier layer on the asphalt surface to improve the flame-retardant performance.

#### 3.2.3. Char Residue Analysis

**Figure 8:**
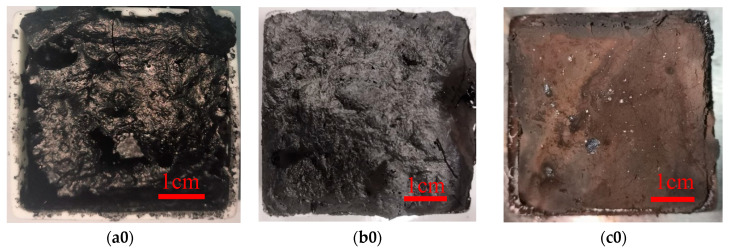

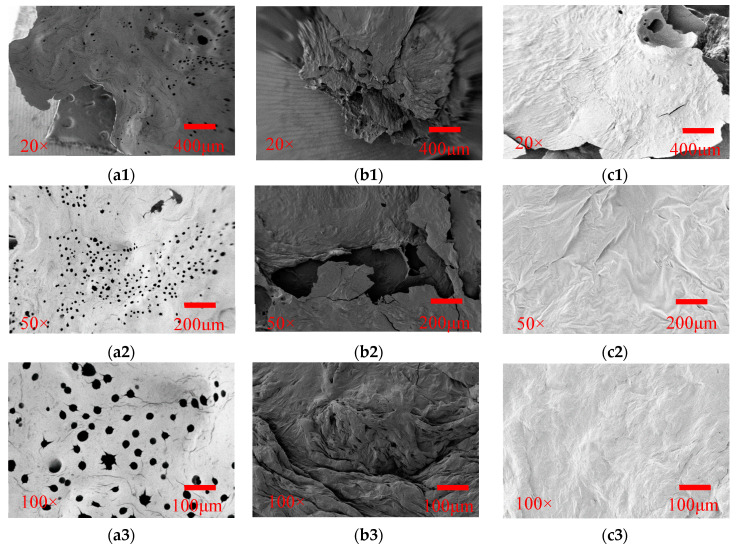
Digital and SEM images of the residues after cone calorimeter tests: (**a**) Sample 2; (**b**) Sample 7; and (**c**) Sample 8. Numbers 0–3 represent digital images and those at 20×, 50×, and 100× magnification, respectively.

According to the above analysis, it is found that the barrier layer formed by the residue may play an important role to improve the flame retardancy performance of asphalt. For this reason, Samples 2, 7, and 8 were heat-treated in a muffle furnace, and then digital cameras and field emission scanning electron microscopes were used to characterize the surface morphology of the residue, as shown in Figure 8.

As can be seen from the first line of Figure 8, there are some carbon shells in Sample 2 after combustion, but the shells are incomplete and discontinuous. With the addition of 8 wt % CFR, there are significantly fewer cracks and holes on the residue surface of Sample 7 than in Sample 2. For Sample 8, with the addition of 1 wt % HNTs/8 wt % CFR, the combustion shell is more compact and complete. This indicates that it is hard for asphalt to form a carbon layer after combustion, and it is also impossible to form a complete barrier layer to prevent further degradation of asphalt. The addition of CFR can increase the amount of residue after combustion and form a relatively complete and dense barrier layer, but there are still some holes and cracks on the surface of the barriers layer. Furthermore, after adding 1 wt % HNTs, the barriers layer is relatively complete and dense from macro perspectives.

The microstructure of the residue was further observed using SEM, as shown in rows 2–4 of Figure 8. It can be found that the residue of Sample 2 is a single thin layer structure, and the surface is distributed with many voids and cracks, which may be caused by the gas generated from the asphalt decomposition. The carbon residue of Sample 7 is a lamellar multilayer structure, but the lamellar structure is not strong enough to cause local collapse. There are many folds in the detailed structure of the residue, which may be caused by the melting flow of fuel. The residue of Sample 8 is also a multilayer flake structure with strong structural integrity. The residue surface is relatively flat without obvious cracks and holes. It is believed that the asphalt flame retardant performance of Sample 2 is not improved after combustion. Adding 8 wt % CFR into asphalt (Sample 7) can effectively prevent further asphalt internal combustion by forming a large number of layer structures. However, due to the poor integrity of the residue, it does not completely cut off the material exchange between the internal material and the combustion interface. Moreover, with the increase of temperature, its structure collapses due to the defect of high-temperature stability, so it is flame retardant properties cannot be fully utilized. Meanwhile, adding 1 wt % HNTs/8 wt % CFR, HNTs plays an important role in enhancing the integrity of silicate layers due to its high-temperature stability. There are no obvious cracks and pores on the surface of the residue structure, thus the residue structure could play the role of the barrier layer and prevent effectively heat and material exchange. Based on these facts, it is suggested that the synergistic effect of CFR and HNTs improves the flame-retardant properties of asphalt.

Based on these facts, the synergistic effect of HNTs and CFR is mainly attributed to the following two points: (1) enhancing the integrity of the residue formed by the combustion of asphalt; and (2) improving the thermal stability and strength of the residue. The complete, dense, and stable residue can serve as a good barrier layer to prevent the heat exchange and material exchange between the asphalt and the combustion zone, thus realizing the flame retardancy effect of asphalt.

### 3.3. Flame Retardant Mechanism of HNTs Coefficient CFR Modified Asphalt

**Scheme 1:**
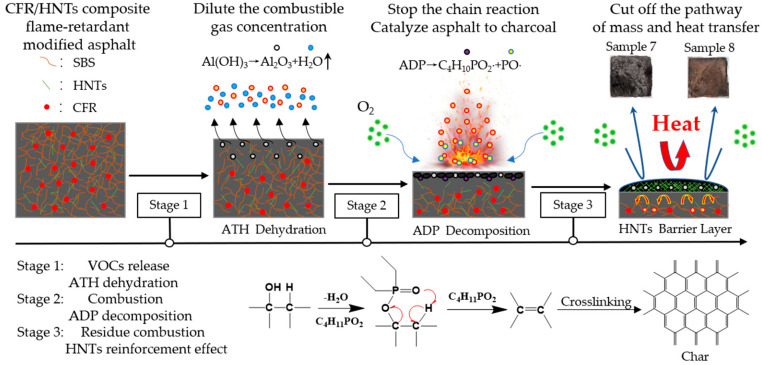
Flame-retardant mechanism of HNTs/CFR flame-retardant system for asphalt.

Based on the above analysis and the comparative study on the change of all-phase material in the combustion process of flame-retardant asphalt, the flame-retardant mechanism of ATH/ADP/HNTs flame retardant-modified asphalt shown in Scheme 1 is analyzed and summarized. Firstly, ATH works as the flame retardant at low temperature (about 220–300 °C) by absorbing heat and lessening the volatilization of lightweight components. At the same time, the decomposition produces water vapor, which dilutes the concentration of flammable gas and inhibits the spread of flame, and forms good thermal stability metal oxide Al_2_O_3_, which improves the thermal stability of the carbon layer and compacts the barrier layer. The flame retardancy effect of ADP is obvious at 380–540 °C. The thermal decomposition of ADP can produce organic phosphoric acid and some volatile free radicals. Organic phosphoric acid can catalyze asphalt crosslinking and ring thickening and increase the rate of asphalt residue generation. Volatile free radicals can capture hydrogen free radicals and hydroxyl free radicals in the flame zone, and terminate the chain reaction and achieve the flame retardancy effect. Except that, HNTs, as a flame retardant, does not significantly improve the flame retardancy performance of asphalt. The main function of HNTs is to act as a synergist of conventional flame retardants. HNTs could significantly enhance the compactness and thermal stability of the barrier layer and improve the flame retardancy efficiency of asphalt. The barrier layer covering the asphalt surface could effectively cut off the heat and material exchange between the asphalt and the combustion flame zone and prevent further degradation. The main feature of HNTs is its obvious synergistic effect, which can significantly reduce the PHRR and PSPR and promote barrier layer formation at a very small amount of addition. However, the HNTs/CFR flame retardant system also has disadvantages. For example, compared with SBS-modified asphalt, its low temperature performance is still degraded, and the preparation process is more complicated, which will increase the cost by about 18%. The effect of flame retardants on the regeneration performance and rheological properties of asphalt will also be studied.

## 4. Conclusions

The flame retardance properties and mechanism of modified asphalt with HNTs and CFR were investigated in this study, and the following conclusions can be drawn:

1. Flame retardancy properties

The flame retardancy properties and synergistic effects of CFR with HNTs in asphalt binder were investigated using IT, LOI, and CCT. The results show that HNTs have an obvious synergistic effect with CFR in improving the flame-retardant properties of asphalt. LOI value could reach 25.5% at the optimum ratio of 1 wt % HNTs/5 wt % ADP/3 wt % ATH. At the same time, the IT value of the HNTs/CFR flame-retardant asphalt system increased by 42 °C. The results of CCT show that the values PHRR, PSPR, and ML of asphalt with HNTs/CFR decreased remarkably compared with those of SBS-modified asphalt and CFR-modified asphalt, decreasing, respectively, from 993.1 to 495.4 kW/m^2^, from 0.279 to 0.211 m^2^/s, and from 96.3% to 89.2%. It is believed that HNT/CFR-modified asphalt system has excellent flame-retardant performance.

2. Flame retardancy mechanism

The TGA and DTG showed that the HNTs/CFR system-modified asphalt could have higher thermal stability and make more residue formation compared with SBS-modified and CFR-modified asphalt. The decomposition rate of asphalt at Stages 2 and 3 decreased obviously. FTIR showed that ATH plays a major role in flame retardation of flame-retardant-modified asphalt by absorbing heat, and Al_2_O_3_ is formed to contribute to the formation of the barrier layer. ADP catalyzes the condensation of asphalt to carbon by producing phosphoric acid and produced free radicals to block the combustion process. HNTs, as the synergist of CFR, plays a reinforcing role in the formation of the barrier layer. SEM results show that a compact and thick barrier layer is formed in HNTs/CFR system-modified asphalt, which hinders the transfer of heat flow and combustible gases at the combustion interface. Thus, the combination of HNTs and CFR has been proved to be a prospective flame-retardant system for asphalt.

## Figures and Tables

**Table 1:** Composition and physical properties of composite flame-retardant-modified asphalt binder.

No.	Matrix Asphalt	SBS wt %	HNTs wt %	ATH wt %	ADP wt %	Penetration/0.1 mm	Softening Point/°C	Ductility/cm
Sample 1	100	-	-	-	-	67.5	49.8	>100 (15 °C)
Sample 2	100	5	-	-	-	51.9	71.5	30 (±5)
Sample 3	100	5	1	-	-	50.8	78.7	35 (±4)
Sample 4	100	5	2	-	-	48.3	82.3	37 (±2)
Sample 5	100	5	-	5	3	42.3	83.6	18 (±3)
Sample 6	100	5	-	4	4	45.6	81.3	24 (±5)
Sample 7	100	5	-	3	5	48.3	80.5	28 (±2)
Sample 8 ^1^	100	5	1	3	5	45.6	87.3	30 (±3)
Sample 9	100	5	2	3	5	42.0	91.2	28 (±2)
Sample 10 ^2^	100	-	-	-	-	64.3	50.6	>100 (15 °C)
Standard						GB/T 0604	GB/T 0606	GB/T 0605

^1^ The values of 3 wt % ATH and 5 wt % ADP were chosen as the best ratio of CFR according to the flame retardancy properties. ^2^ The composition of Sample 10 is the same as that of Sample 1, but it went through the process shown in Figure 2 without adding flame retardant materials.

**Table 2:** Flammability characteristics obtained from cone calorimeter tests.

No.	TTI (s)	PHRR (kw/m^2^)	THR (MJ/m^2^)	PSPR (m^2^/s)	TSR (m^2^)	ML (%)
Sample 2	21	993.1	239.1	0.279	43.2	96.30
Sample 7	28	666.6	170.3	0.231	41.3	92.04
Sample 8	30	495.4	128.1	0.211	40.1	89.20

## Abbreviations

HNTs	halloysite nanotubes
CFR	conventional flame retardants
COC	Cleveland open cup method
LOI	Limiting oxygen index
CCTs	Cone calorimeter tests
TGA	Thermogravimetric Analyzer
FTIR	Fourier-transform Infrared
SEM	Scanning Electron Microscopy
ATH	Aluminum hydroxide
ADP	Aluminum diethyl phosphate
Al_2_O_3_	Aluminum oxide
OLSs	organic layered silicates
OMMT	organic montmorillonite
VOCs	Volatile organic compounds
OEVMT	organic expanded vermiculite
LDHs	nano-layered hydroxides
EG	expanded graphite
CEPPA	2-carboxy ethyl phenyl phosphonic acid
IT	ignition temperature
TTI	time to ignition
PHRR	the peak of heat release rate
PSPR	the peak of smoke produce rate
THR	total heat release
TSR	total smoke production
ML	mass loss
